# Hand hygiene compliance: bridging the awareness-practice gap in sub-Saharan Africa 

**DOI:** 10.3205/dgkh000341

**Published:** 2020-05-06

**Authors:** Jahmai Irehovbude, Chukwuemeka A. Okoye

**Affiliations:** 1 Department of Human Kinetics and Health Education, Ambrose Alli University, Ekpoma, Edo State, Nigeria; 2 Department of Pathology, Federal Medical Centre Asaba, Delta State, Nigeria

**Keywords:** hand hygiene, multimodal strategy, skill-based hygiene education, sub-Saharan Africa

## Abstract

This review provides an exploratory overview of hand hygiene compliance in sub-Saharan Africa and examines strategies to bridge the compliance gap.

While there is increasing awareness on hand hygiene, empirical evidence suggests that there is no concurrent increase in correct hand hygiene practice among key populations in sub-Saharan Africa. Children, adolescents and even healthcare providers (HCPs) in sub-Saharan Africa consistently assume poor hand hygiene compliance levels resulting in negative health consequences. Faecal-oral diseases remain common among schoolchildren, leading to school absenteeism and disease-specific morbidity. Additionally, the incidence of nosocomial infections in health facilities in sub-Saharan Africa remains high, as many HCPs do not adopt good hand hygiene practice. Increased disease burden, high healthcare costs and eroding public confidence in the healthcare system are a few implications of HCPs’ poor compliance with hand hygiene. These trends underscore the inadequacies of educational approaches (cognition model) to hand hygiene promotion commonly adopted in sub-Saharan Africa.

It was therefore recommended that the governments of sub-Saharan Africa should focus on promoting skill-based hygiene education which will help schoolchildren develop good hand hygiene practice as a lifelong skill. In addition, efforts should be made to implement a multimodal hand hygiene strategy in healthcare facilities in order to increase compliance by healthcare providers.

## Zusammenfassung

Die Übersicht gibt einen explorativen Überblick über die Einhaltung der Händehygiene in Afrika südlich der Sahara und untersucht Strategien zur Überbrückung der Compliance-Lücke.

Während das Bewusstsein für die Händehygiene zunimmt, weisen empirische Daten darauf hin, dass es in den Hauptbevölkerungsgruppen in Afrika südlich der Sahara keine übereinstimmende Zunahme der korrekten Händehygiene gibt. Bei Kindern und Jugendlichen, aber auch bei Mitarbeitern in Gesundheitseinrichtungen (GE) in Afrika südlich der Sahara ist durchweg von einer schlechten Händehygiene auszugehen, was sich negativ auf die Gesundheit auswirkt. Fäkal-orale Erkrankungen treten bei Schulkindern weiterhin häufig auf, was zu Fehlzeiten und krankheitsspezifischer Morbidität führt. Darüber hinaus ist die Inzidenz nosokomialer Infektionen in Gesundheitseinrichtungen in Afrika südlich der Sahara nach wie vor hoch, da viele GE keine gute Handhygienepraxis anwenden. Erhöhte Krankheitslast, hohe Gesundheitskosten und schwindendes Vertrauen der Öffentlichkeit in das Gesundheitssystem sind nur einige Folgen der schlechten Einhaltung der Händehygiene durch die GE. Diese Trends unterstreichen die Unzulänglichkeiten der pädagogischen Ansätze (Kognitionsmodell) zur Förderung der Händehygiene in Afrika südlich der Sahara.

Als Schlussfolgerung wird die Notwendigkeit einer kompetenzbasierten Hygieneausbildung abgeleitet, die Schülern helfen soll, eine gute Handhygienepraxis als lebenslange Fertigkeit zu entwickeln. Für GE steht die Umsetzung einer multimodalen Strategie im Krankenhausumfeld im Mittelpunkt, um die Compliance zu verbessern.

## Introduction

Over the years, hand hygiene (HH) has been a topical issue within the public health discussion in sub-Saharan Africa. This is because of its recognized importance as the cornerstone of infection prevention and control [1]. Infectious diseases are the major cause of morbidity and mortality in sub-Saharan Africa. Worldwide, about 2 million children – 5 years or younger – die from diarrhoea and acute lower respiratory tract infections annually [2]. Many of these deaths occur among children in sub-Saharan Africa [3]. Additionally, healthcare-associated infection (HAI) is a major concern in healthcare delivery, especially in sub-Saharan Africa. Countries within this region – as part of the community of developing nations – report HAI up to 20 times higher than in developed countries [4]. Healthcare-associated infections account for prolonged hospital stays and increased health care costs, contributing significantly to the indices of morbidity and mortality. Millions of deaths can be prevented annually by good hygiene practice, reliable sanitation and drinking water [5].

According to the World Health Organisation, HH is a general term referring to any action of hand cleansing with the use of soap and water or any other liquid, to remove soil, dirt, and/or microorganisms [6]. The washing of hands with soap and water is the most common hand hygiene practice in households and schools. Nevertheless, besides the use of soap and water for hand cleansing, alcohol-based hand rubs (ABHR) are often used in the healthcare setting.

Hitherto, educational interventions are the commonly adopted strategy for increasing HH awareness and compliance in sub-Saharan African. Personal hygiene is usually taught in primary schools to help pupils acquire appropriate knowledge and adopt healthful behaviour, such as hand hygiene. Awareness on HH has also grown with the help of targeted HH awareness programmes, promoted through the mass media. Additionally, healthcare providers (HCPs) usually demonstrate a high awareness level of hand hygiene, as HH – being part of healthcare safety – is a key thematic area in the education of many HCPs.

Sadly, despite these initiatives, HH compliance in sub-Saharan Africa remains stubbornly low [7]. A didactic or educational approach to HH promotion alone produces insufficient outcomes. While such interventions can help create awareness, improve knowledge, and clarify misconceptions, they often do not result in the behavioural change that engenders long-term HH compliance.

## Hand hygiene practice among schoolchildren in sub-Saharan Africa

The early school years are formative in the life of an individual. During this period, lifelong habits are learned. Thus, early school age is seen as the most important period for turning HH rules into behaviour [8]. Proper HH among schoolchildren in sub-Saharan Africa is generally low, and educational interventions with a focus on the cognitive domain are usually adopted to improve HH among this population. A survey of the knowledge, attitudes and practice of hand hygiene among schoolchildren in Kintampo, Ghana, showed that only 37.7% of the schoolchildren (n=300) claimed to wash their hands with soap and water [9]. However, only 23.3% of the children demonstrated correctly how to wash hands [9]. In a cross-sectional study involving 426 children in six primary schools in Ghana, Mekonnen, Aga, Kinati, and Shifera reported that only 32% of the study population washed their hands properly with soap and water before meals and after visiting the toilet [10]. It was observed that students from rural areas were less likely to wash their hands properly in comparison to students from urban areas [10]. Whether this was attributable to the unavailability of water and soap in their environment or due to other factors such as lack of education is not clear.

A study on the effectiveness of handwashing education on primary school pupils in eastern Nigeria showed that there was increased knowledge on handwashing in the intervention group (x=10.87%) than in the comparison group (x=0.08%) who did not receive the educational intervention [11]. It was also reported that the post-intervention handwashing skill of the intervention group (x=12.81%) was greater than the comparison group (x=4.40%) [11]. In a cluster randomised controlled trial involving Malawian schoolchildren, Mbakaya et al. [12] reported a higher knowledge score among the intervention group than in the control group after baseline. They also noted that the technique score in the intervention group was significantly higher than the control group after the intervention. While these studies show the gains made in increasing HH awareness, knowledge, and practice among schoolchildren through education-based HH interventions, it should be noted that self-reported data on HH hygiene compliance are often unreliable [13]. Thus, studies based on self-reported HH data after an educational intervention may tend to over-report HH compliance. Observational studies may provide a more accurate picture of the HH practices of schoolchildren and their long-term adherence to HH post-intervention.

## Health care providers’ hand hygiene compliance in sub-Saharan Africa

Hand hygiene – essential in the prevention and reduction of healthcare-associated infection (HAI) – is poor among HCPs in sub-Saharan Africa [1],[14] , [15], [16], [17]. Patients and HCPs alike are at risk of nosocomial infections. Healthcare-associated infection is estimated to affect about 5–15% of all hospitalized patients and a greater percentage (30%) of patients in intensive care units (ICUs) in sub-Saharan Africa [18].

The hands of HCPs are an important vehicle for the transmission of nosocomial pathogens [19]. Poor HH by HCPs is responsible for about 40% of nosocomial infections worldwide [20]. Prevalence studies of nosocomial infection in sub-Saharan Africa have reported higher rates than other parts of the world [21], [22].

A study by Jemal [23] of the knowledge and practice of HH among HCPs in a referral hospital in Northeast Ethiopia showed that the majority of the HCPs (65.9%) were knowledgeable. However, most of the HCPs (56%) had poor HH practice. A similar study of the practice of handwashing among nurses in a general hospital in Akwa Ibom State, Nigeria, showed that 82.4% of the nurses had good knowledge of handwashing, whereas only 42.2% of the nurses regularly practised handwashing [24]. Similarly, in the study of HCPs’ HH practice in a teaching hospital in Ghana, Yawson and Hesse [25] reported that HH compliance ranged between 9.2% to 57% among doctors and 9.6% to 54% among nurses. Shobowale et al. [1] monitored the HH compliance of healthcare workers in a teaching hospital in Nigeria using the “Five Moments for Hand Hygiene” guideline of the WHO. Results showed that HH compliance at the five key moments was unacceptably low among the healthcare workers.

The foregoing shows that good awareness and knowledge of HH correlates poorly with behavioural change. The use of an educational or didactic approach alone is often insufficient in promoting behavioural change in HH practice among schoolchildren and even HCPs. As noted by Shobowale et al. [1], HCPs may “see educational programmes as mundane”. There is a need to focus on evidence-based approaches that can help overcome HH barriers, promote behavioural change, be applicable even in settings where resources are limited, and scalable.

## Evidence-based approaches to hand hygiene promotion

In the promotion of HH compliance both in schools and hospital settings, the use of multi-level strategies has shown much promise in improving HH compliance among children, adolescents, and HCPs alike. A multi-level strategy involves the use not only of education but other nudges that help increase the self-efficacy of individuals to practice HH.

### Skill-based hygiene education in schools

Skill-based health education focuses on the development of knowledge, attitudes, values and skills, including life skills such as interpersonal skills and decision making, which are needed to make and act on the most appropriate and positive health-related decision. It allows students to practise the skills they need to avoid risky and unhealthy situations and adopt and sustain healthier lifestyles. Skill-based health education is an essential component of the school health programme, as it increases the self-efficacy of students. Evidence shows that intervention programmes which target social norms and self-efficacy are most effective in promoting HH among schoolchildren [8], [26]. Hygiene education – being part of health education – that is skill-based empowers children and adolescents to develop the life skills they need to stay healthy. It enables people to become innovative and to improvise even in resource-limited settings where water is scarce or in short supply. As part of skill-based hygiene education, schoolchildren can be trained to develop tippy taps in a setting where water is scarce. A tippy tap is a cheap hands-free HH promotion facility that helps avoid the recontamination of hands possible in the use of regular taps. Even in very poor rural areas, the creation and use of tippy taps have proven to be effective. In a school-based HH intervention study by Zhang et al. [27], schoolchildren in Uganda were assigned to an intervention group (n=200) and a control group (n=195). The control group were given regular HH education, while the intervention group not only received HH education but were also trained on the construction and maintenance of tippy taps and had tippy taps provided as part of the intervention. Post-intervention, only 11.3% of the control group reported washing their hands either “always” or “often”, whereas 100% of the intervention group reported washing their hands “always” or “often”. This study affirms that skill-based HH education is more effective at influencing HH compliance among schoolchildren than traditional HH education. Sub-Saharan African countries should focus on providing HH education that is skill-based to children within this region to increase HH compliance among this population.

### Multimodal strategy in the health care system 

A multimodal strategy is recommended in the WHO guideline for HH in healthcare to reduce HAI [6]. It is the most effective means yet to improve HH compliance among HCPs and reduce HAI [28], [29], [30], [31]. The implementation of the multimodal strategy in a tertiary hospital in Taiwan increased HH compliance among HCPs from 62.3% to 73.3% after a year [30]. A decrease in HAI (from 3.7%to 3.1%) was observed, and the campaign was estimated to have saved an estimated $940,000 and 3,564 admission patient days per year.

The promotion of HH through a multimodal strategy is feasible even in resource-constrained settings, and its adoption can increase HH compliance among HCPs even in small rural hospitals [31], [32], [33]. An integrative review of twenty-five studies, in which a multimodal strategy was adopted in the healthcare setting, showed that a multimodal strategy improved hand hygiene compliance and ensured its long term sustainability [28].

**Figure 1:**
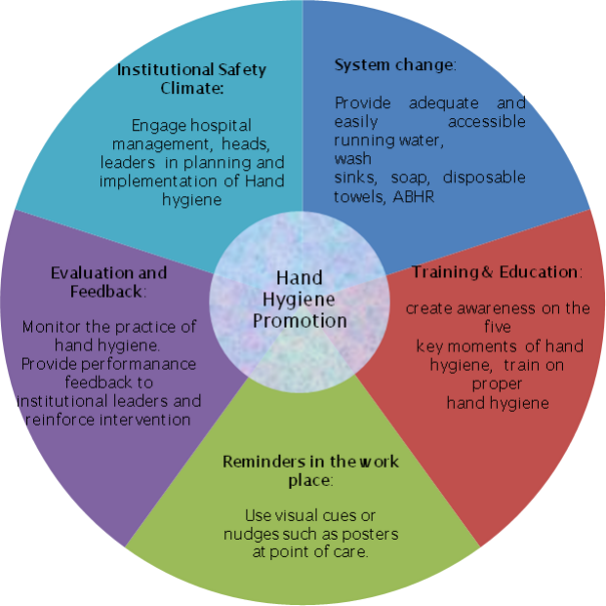
The five components of a multimodal strategy

The WHO introduced the concept of the “Five Moments for Hand Hygiene”, which comprise the moment before touching a patient, before performing an aseptic and clean procedure, after being at risk of exposure to body fluids, after touching a patient, and after touching patient surroundings [6]. Compliance with hand hygiene at these key moments can be attained through the five components of a multimodal strategy (Figure 1 (Fig. 1)) which are:

- System change: This involves the provision of the critical infrastructure that will enable HCPs to perform hand hygiene. This critical infrastructure includes the provision of running water, soaps, disposable towels and ABHR at each point of care.
- Training/education: A user-centred education and training of HCPs on the need for hand hygiene, the “Five Moments for Hand Hygiene”, and the correct procedure for handwashing and rubbing.
- Evaluation and feedback: Continuous monitoring of the indicators for hand hygiene compliance including the available HH infrastructure and incidence of HAI within the health care setting. The use of covert observers in place of overt observers is more effective as it decreases the observer’s bias [34].
- Reminders in the workplace: This comprises all the tools to prompt and remind HCPs of the need for HH, including the use of visual cues such as posters at point of care, for example the utilisation of the WHO toolkit on the “Five Moments for Hand Hygiene” [6]. The positioning of HH facilities in conspicuous locations at point of care can serve as useful visual cues.
- Institutional safety climate: This entails the engagement of all relevant stakeholders – decision-makers, leaders, managers, heads, and influential HCPs – within the healthcare setting in planning the HH programme and at stages of its implementation. This will help garner adequate support for the programme. These five components of a multimodal strategy can be implemented within any healthcare institution, even in resource-limited settings [28].

## Conclusions

While some gains have been made in the control of infectious diseases in sub-Saharan Africa, the challenge of infectious diseases among schoolchildren and nosocomial infections still looms large. Educational interventions – common in sub-Saharan Africa – have increased awareness on HH, but such interventions alone cannot induce the needed behavioural changes for HH compliance. Multi-dimensional approaches, such as a focus on skill-based hygiene education and the adoption of multimodal strategies, can help bridge the awareness-practice gap in HH in sub-Saharan Africa. In conclusion, the following recommendations are given:

1. Schoolteachers should be trained to provide hygiene education that is skill-based to student.
2. Students should be trained on proper hand washing procedure and hand wash facilities should be provided by the school authorities.
3. In school settings where water is scarce, tippy taps should be deployed by the school authorities to encourage hand hygiene among students.
4. Students should be trained – as part of skill-based hygiene education – on the procedures for making their own tippy taps for hand hygiene practice at home.
5. In the healthcare setting, efforts should be made by health institutions to strengthen any educational intervention on HH by adopting a multimodal strategy.
6. Hand hygiene facilities such as liquid soap, running water and health facilities alcohol-based hand rub should be readily available within point of care.
7. Visual clues should be provided to promote hand hygiene compliance especially at point of care
8. Compliance of HCPs with hand hygiene in health care institutions should be ensured through inclusion of hand hygiene in standard operating procedures. Where appropriate, covert observation of staff should also be adopted.
9. The governments of sub-Saharan Africa countries should make policies – within their national context – that promote skill-based hygiene education and the multimodal strategy in schools and health care institutions, respectively.

## Notes

### Competing interests

The authors declare that they have no competing interests.
